# Draft genome sequence of *Staphylococcus epidermidis* strain UMP2601 isolated from environmental water

**DOI:** 10.1128/mra.00479-26

**Published:** 2026-08-18

**Authors:** Norafisah Mohd Arshad, Siti Khadijah Kiraman, Saidatul Akasha Sufian, Hajar Fauzan Ahmad

**Affiliations:** 1 Faculty of Industrial Sciences and Technology, Universiti Malaysia Pahang Al-Sultan Abdullah 65240, Gambang, Pahang, Malaysia; Loyola University Chicago, Chicago, Illinois, USA

**Keywords:** skin microbiome

## Abstract

We report the draft genome sequence of *Staphylococcus epidermidis* strain UMP2601 (2,469,061 bp, GC content of 32.03%) isolated from an environmental water sample. The organism was cultured on nutrient agar under aerobic conditions.

## ANNOUNCEMENT

*Staphylococcus epidermidis,* a coagulase-negative *Staphylococcus*, is a key component of the skin microbiota and is linked to healthcare-related infections (1). It can become opportunistic, particularly through biofilm formation, which enhances its persistence and resistance to antimicrobials (2). We report the draft genome of *S. epidermidis* strain UMP2601 isolated from a surface freshwater sample collected from puddles near Tasik Paya Besar, Gambang, Pahang, Malaysia (GPS: 3.7070° N, 103.1025° E) in January 2026.

The water sample was serially diluted 10-fold in autoclave-distilled water and plated on nutrient agar, then incubated overnight at 37°C (3). A white, smooth colony was isolated, cultured in nutrient broth, and genomic DNA was extracted with the NucleoSpin Microbial DNA Mini Kit (Macherey-Nagel, Germany) according to the manufacturer’s protocol. Libraries were constructed using the Illumina DNA Prep Kit (Illumina, San Diego, CA, USA) and sequenced on the Illumina HiSeq X platform (2 × 150 bp paired-end).

Reads were processed with BBDuk v38.90 (4) to remove adapters and low-quality sequences. SPAdes v3.15.3 (5) was run in short-read assembly mode using paired-end Illumina reads to generate contigs and scaffolds. Assemblies were polished using Pilon v1.24 (6) with mapped Illumina reads. Assembly quality was assessed using QUAST v5.3.0 (7). Plasmid and phage sequences were identified using geNomad v1.11.2 (8). Multilocus sequence typing (MLST) was performed using mlst v2.32.2 (9) based on the PubMLST scheme to assess the allelic profile. Assembly completeness was assessed using BUSCO v6.0.0 (10) with the staphylococcus_odb12 data set. Protein-coding genes were predicted using Prodigal v2.6.3 (11) and functionally annotated with eggNOG-mapper v2.1.13 (12). rRNA and tRNA gene counts were obtained from NCBI PGAP v6.11 (13).

Antimicrobial resistance genes and virulence factors were screened using ABRicate v1.2.0 (14) against the CARD, NCBI, ResFinder, VFDB, and Victors (15) databases dated 7 April 2026. Default parameters were used unless otherwise specified.

geNomad v1.11.2 (8) predicted several high-confidence plasmid-associated contigs and multiple prophage regions, including sequences affiliated with *Caudoviricetes*. mlst v2.32.2 (9) analysis placed the isolate within the *S. epidermidis* scheme, although no existing sequence type was assigned, as shown in Table 1. Antimicrobial resistance genes were identified. The genome harbors a complete beta-lactam resistance system, including *blaZ*, *blaR1*, and *blaI* (16), as well as *tet(K)* (17), indicating resistance to beta-lactam and tetracycline antibiotics. Additional genes such as *dfrS1* and *fosB* suggest potential resistance to trimethoprim (18) and fosfomycin (19), whereas no virulence factors were detected in the VFDB database.

**TABLE 1:** Genome characteristics of *Staphylococcus epidermidis* strain UMP2601

Parameter	Result
Total number of reads	7,007,128
Genome size (bp)	2,469,061
GC content (%)	32.03
Genome coverage	212.8×
Number of contigs	81
N50	71,206
Number of predicted genes	2,375
rRNA	3
tRNA	47
MLST	*S. epidermidis* scheme; no ST assigned; 7/7 loci recovered; 6 exact matches (arcC-12, aroE-29, gtr-9, mutS-8, tpiA-5, yqiL-8) and 1 non-exact match (pyrR~6)
BUSCO data set	
Complete BUSCOs (C)	98.1%
Single-copy BUSCOs (S)	97.8%
Duplicated BUSCOs (D)	0.3%
Fragmented BUSCOs (F)	0.2%
Missing BUSCOs (M)	1.7%
Total BUSCO groups searched (*n*)	1,317

## Data Availability

This Whole Genome Shotgun project has been deposited at DDBJ/ENA/GenBank under accession number JBWXXE000000000. The version described in this paper is version JBWXXE010000000. The sequencing data are available in the SRA under accession number SRR37905578, associated with BioProject PRJNA1447422 and BioSample SAMN56964660.

## References

[1] Becker K, Both A, Weißelberg S, Heilmann C, Rohde H. 2020. Emergence of coagulase-negative staphylococci. Expert Rev Anti Infect Ther 18:349–366. doi:10.1080/14787210.2020.1730813 32056452

[2] França A. 2023. The role of coagulase-negative staphylococci biofilms on late-onset sepsis: current challenges and emerging diagnostics and therapies. Antibiotics 12:e554. doi:10.3390/antibiotics12030554 PMC10044083 36978421

[3] Ahmad HF, Schreiber L, Marshall IPG, Andersen PJ, Castro-Mejía JL, Nielsen DS. 2019. Draft genome sequence of *Streptococcus anginosus* strain CALM001, isolated from the gut of an elderly dane. Microbiol Resour Announc 8:e00379-19. doi:10.1128/MRA.00379-19 31196919 PMC6588037

[4] Bushnell B. 2014. BBMap: a fast, accurate, splice-aware aligner. 38.90. Lawrence Berkeley National Laboratory.

[5] Bankevich A, Nurk S, Antipov D, Gurevich AA, Dvorkin M, Kulikov AS, Lesin VM, Nikolenko SI, Pham S, Prjibelski AD, Pyshkin AV, Sirotkin AV, Vyahhi N, Tesler G, Alekseyev MA, Pevzner PA. 2012. SPAdes: a new genome assembly algorithm and its applications to single-cell sequencing. J Comput Biol 19:455–477. doi:10.1089/cmb.2012.0021 22506599 PMC3342519

[6] Walker BJ, Abeel T, Shea T, Priest M, Abouelliel A, Sakthikumar S, Cuomo CA, Zeng Q, Wortman J, Young SK, Earl AM. 2014. Pilon: an integrated tool for comprehensive microbial variant detection and genome assembly improvement. PLoS One 9:e112963. doi:10.1371/journal.pone.0112963 25409509 PMC4237348

[7] Gurevich A, Saveliev V, Vyahhi N, Tesler G. 2013. QUAST: quality assessment tool for genome assemblies. Bioinformatics 29:1072–1075. doi:10.1093/bioinformatics/btt086 23422339 PMC3624806

[8] Camargo AP, Roux S, Schulz F, Babinski M, Xu Y, Hu B, Chain PSG, Nayfach S, Kyrpides NC. 2024. Identification of mobile genetic elements with geNomad. Nat Biotechnol 42:1303–1312. doi:10.1038/s41587-023-01953-y 37735266 PMC11324519

[9] Seemann T. 2023. mlst. 2.32.2. GitHub

[10] Manni M, Berkeley MR, Seppey M, Zdobnov EM. 2021. BUSCO: assessing genomic data quality and beyond. Current Protocols 1:e323. doi:10.1002/cpz1.323 34936221

[11] Hyatt D, Chen G-L, Locascio PF, Land ML, Larimer FW, Hauser LJ. 2010. Prodigal: prokaryotic gene recognition and translation initiation site identification. BMC Bioinformatics 11:e119. doi:10.1186/1471-2105-11-119 PMC2848648 20211023

[12] Cantalapiedra CP, Hernández-Plaza A, Letunic I, Bork P, Huerta-Cepas J. 2021. eggNOG-mapper v2: functional annotation, orthology assignments, and domain prediction at the metagenomic scale. Mol Biol Evol 38:5825–5829. doi:10.1093/molbev/msab293 34597405 PMC8662613

[13] Tatusova T, DiCuccio M, Badretdin A, Chetvernin V, Nawrocki EP, Zaslavsky L, Lomsadze A, Pruitt KD, Borodovsky M, Ostell J. 2016. NCBI prokaryotic genome annotation pipeline. Nucleic Acids Res 44:6614–6624. doi:10.1093/nar/gkw569 27342282 PMC5001611

[14] Seeman T. 2025. ABRicate: mass screening of contigs for antimicrobial resistance or virulence genes. GitHub

[15] Sayers S, Li L, Ong E, Deng S, Fu G, Lin Y, Yang B, Zhang S, Fa Z, Zhao B, Xiang Z, Li Y, Zhao XM, Olszewski MA, Chen L, He Y. 2019. Victors: a web-based knowledge base of virulence factors in human and animal pathogens. Nucleic Acids Res 47:D693–D700. doi:10.1093/nar/gky999 30365026 PMC6324020

[16] Wang P-Z, Projan SJ, Novick RP. 1991. Nucleotide sequence of β-lactamase regulatory genes from staphylococcal plasmid p1258. Nucl Acids Res 19:4000–4000. doi:10.1093/nar/19.14.4000 1861992 PMC328499

[17] Yamaguchi A, Shiina Y, Fujihira E, Sawai T, Noguchi N, Sasatsu M. 1995. The tetracycline efflux protein encoded by the tet(K) gene from *Staphylococcus aureus* is a metal-tetracycline/H+ antiporter. FEBS Lett 365:193–197. doi:10.1016/0014-5793(95)00455-i 7781778

[18] Lombardo MN, G-Dayanandan N, Wright DL, Anderson AC. 2016. Crystal structures of trimethoprim-resistant DfrA1 rationalize potent inhibition by propargyl-linked antifolates. ACS Infect Dis 2:149–156. doi:10.1021/acsinfecdis.5b00129 27624966 PMC5108240

[19] Wiltsie V, Travis S, Shay MR, Simmons Z, Frantom P, Thompson MK. 2022. Structural and functional characterization of fosfomycin resistance conferred by FosB from *Enterococcus faecium*. Protein Sci 31:580–590. doi:10.1002/pro.4253 34882867 PMC8862413

